# Wolman disease presenting with hemophagocytic lymphohistiocytosis syndrome and a novel LIPA gene variant: a case report and review of the literature 

**DOI:** 10.1186/s13256-023-04116-4

**Published:** 2023-08-29

**Authors:** Kosar Asna Ashari, Aileen Azari-Yam, Mohammad Shahrooei, Vahid Ziaee

**Affiliations:** 1 Children’s Medical Center, Pediatrics Center of Excellence, Dr Qarib St, Keshavarz Blvd, Tehran, 14194 Iran; 2 Department of Pediatrics, Tehran University of Medical Sciences, Tehran, Iran; 3 Pediatric Rheumatology Society of Iran, Tehran, Iran; 4 Pediatrics Rheumatology Research Group, Rheumatology Research Center, Tehran University of Medical Sciences, Tehran, Iran; 5 Department of Microbiology and Immunology, Laboratory of Clinical Bacteriology and Mycology, KU Leuven, Leuven, Belgium

**Keywords:** Wolman disease, Hemophagocytic lymphohistiocytosis, HLH, *LIPA* gene

## Abstract

**Background:**

Wolman disease is a rare disease caused by the absence of functional liposomal acid lipase due to mutations in *LIPA* gene. It presents with organomegaly, malabsorption, and adrenal calcifications. The presentations can resemble hemophagocytic lymphohistiocytosis, the life threatening hyperinflammatory disorder. Since the disease is very rare, clinicians might not think of it when a patient presents with hemophagocytic lymphohistiocytosis, and the opportunity to treat it properly can be lost, thus leading to demise of the child.

**Case presentation:**

We present a 4.5-month-old Caucasian boy with fever, icterus, and hepatosplenomegaly who was treated according to presumed hemophagocytic lymphohistiocytosis disease. Wolman disease was diagnosed after the death of the child. There are some case reports in the literature presenting patients with Wolman disease primarily diagnosed as hemophagocytic lymphohistiocytosis, which we discuss in this review. The genetic analysis revealed after his demise was compatible with Wolman disease, introducing a novel mutation in *LIPA* gene: exon 4: NM_001127605: c. G353A (p.G118D), which converts the glycine amino acid to aspartic acid.

**Conclusions:**

Considering the similarities in presentation of Wolman disease and hemophagocytic lymphohistiocytosis, the patient’s life can be saved if special attention is paid to presenting features of a patient with suspected hemophagocytic lymphohistiocytosis, that is special attention to symptoms, findings on physical exams, laboratory values, and radiologic findings, and the proper treatment is urgently initiated. Reporting the novel mutations of Wolman disease can help geneticists interpret the results of their patients’ genetic studies appropriately, leading to correct diagnosis and treatment.

## Background

Hemophagocytic lymphohistiocytosis (HLH) is a life threatening hyperinflammatory disorder, mostly described in children, but indeed affecting all ages. The pathogenesis is rooted in deregulated immune homeostasis. Uncontrolled proliferation of activated lymphocytes and macrophages poses a dramatic hyperinflammatory response, provoking the symptoms and signs associated with the disorder [1]. Fever, hepatosplenomegaly, pancytopenia, hypertriglyceridemia and/or hypofibrinogenemia, high ferritin, large or absent natural killer (NK) cell activity, and increased amount of soluble CD25 are characteristic features of HLH [2, 3].

HLH is classified as familial and secondary. Primary, congenital, or familial type, mostly described in infants, is caused by mutations in genes such as *PRF1*, *MUNC13-4*, and *STX11*. The secondary type is found to be associated with infections, malignancies, immune deficiencies, rheumatologic diseases, and metabolic diseases, including Gaucher disease, lysinuric protein intolerance, and lysosomal enzyme deficiencies [4]. Primary HLH is basically treated by immune suppression which is provided by steroids and chemotherapy. Cure for the condition might be achieved by allogeneic hematopoietic stem cell transplantation (HSCT). Treatment for secondary HLH should primarily trigger the underlying condition, such as infection. The use of immunosuppressives and chemotherapy might also be helpful. New therapies to target immune system are suggested for both types of HLH. The condition is overall fatal and therapies should be initiated as soon as possible to rescue the patient, if possible [1, 5].

Lysosomal storage disease includes a wide array of disorders originating in a defect in lysosomal enzymes or proteins. This leads to the impotency in degradation of proteins and lipids and their accumulation in different sites, causing presentations of various diseases, such as Niemann–Pick, mucopolysaccharidosis, Pompe, cholesteryl ester storage disease (CESD), and Wolman disease (WD).

WD is rare and appears in less than 1 in 100,000 newborns. It is induced by the absence of functional liposomal acid lipase (LAL), caused by mutations in *LIPA* genes. The disease is characterized by accumulation of foamy lipid droplets in lysosomes of tissues such as liver, spleen, intestine, and lymph nodes. The patient presents with hepatosplenomegaly, failure to thrive, malabsorption, and organ specific symptoms such as hepatic damage. Adrenal calcification signifies a unique picture in WD [6]. Enzyme replacement therapy with sebelipase alfa has made a better prognosis available for patients with WD. HSCT and liver transplantation were suggested to be beneficial before the introduction of enzyme replacement therapy [7].

The clinical picture and laboratory findings of HLH and WD overlap to a large extent and that is why in many cases the definite diagnosis cannot be made until genetic analysis results are announced. If WD is an option when the patient is referred with signs of secondary HLH, prompt treatment with enzyme replacement might result in a higher survival rate. We present a 4.5-month-old boy with fever, icterus, and hepatosplenomegaly, with overlapping features of both HLH and WD, and genetic analysis findings compatible with WD. A few studies are accessible in the literature introducing Wolman patients presented with secondary HLH. We review the data in hope for better understanding WD and how it can be related to or differentiated from HLH.

## Case presentation

A 4.5-month-old Caucasian boy was admitted to the hospital with a 2-week history of fever following vaccination and 5 days of jaundice and drowsiness. There was no complaint of vomiting, diarrhea, convulsions or any other neurologic presentations. The parents declared that abdominal distension had been noticeable since birth. He was the only child of first cousins and was born by normal delivery after a 40-week gestation (birth weight: 3750 g, percentile: 61, *z* score: 0.29; birth height: 51 cm, percentile: 26, *z* score: −0.65; birth head circumference: 36 cm, percentile: 62, *z* score: 0.31). Neonatal jaundice was not announced. At 4 months, the infant had weighed 8500 g and by admission he weighed 8000 g.

There was not any significant family history except for leukemia in the patient’s maternal uncle who died at the age of 17.

On examination, the patient appeared pale, icteric, and ill and had mild respiratory distress. The abdomen appeared severely distended and enlarged spleen and liver were found by palpation. No rash was observed and no lymphadenopathy could be found.

**Fig. 1:**
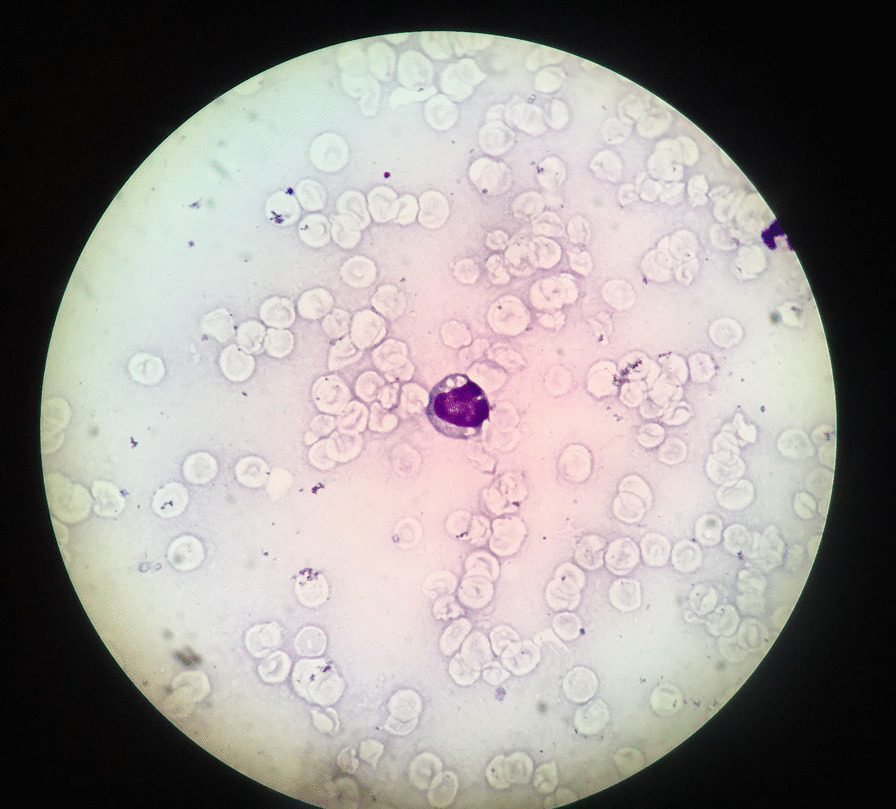
Peripheral blood smear of our patient stained by Romanovski method showing a lymphocyte with cytoplasmic vacuolation

Upon admission the patient had abnormal coagulation tests, elevated liver enzymes, direct hyperbilirubinemia, low albumin, and high ferritin values (Table 1). He was anemic from the beginning, and values of platelet, neutrophils, and lymphocytes that were normal gradually decreased during admission. Figure 1 shows the peripheral blood smear of our patient stained by Romanovski method.

**Table 1:** Patient laboratory findings on admission and on last check

Laboratory values	On admission	Last check
Hemoglobin(g/l)	7.6	4.6
Platelet (× 10^9^/l)	65	19
WBC (× 10^6^/l)	7050	820
Neutrophils (× 10^6^/l)	3730	10
Triglyceride (mg/dl)	1110	–
Cholesterol (mg/dl)	268	–
Ferritin (ng/ml)	5370	2400
LDH (U/l)	2690	–
PT	35	20
PTT	100	36
AST (U/l)	2930	870
ALT (U/l)	603	320
ALP (U/l)	521	351
Albumin (g/dl)	2.8	2.6
NK cell activity	Not done	Not done
Soluble CD25	Not done	Not done

*WBC* white blood cells, *PT* prothrombin time, *PTT* partial thromboplastin time, *AST* aspartate transaminase, *ALT* alanine transaminase, *ALP* alkaline phosphatase

Attempts to find a septic source, including Epstein–Barr virus (EBV) and cytomegalovirus (CMV) polymerase chain reactions (PCRs) and blood culture were unsuccessful. Hypertension was observed and worked up; echocardiography and renal artery sonography were normal. Bone marrow aspiration was nonspecific. Due to primary suspicion of HLH, cyclosporine and dexamethasone were prescribed. Methylprednisolone pulses and intravenous immunoglobulin (Ig) were also tried. This treatment is approved by ethics committee of Children’s Medical Center, Tehran, Iran. Unfortunately, the patient died with a clinical picture of cardiopulmonary compromise due to hepatic failure 17 days after admission. Results of molecular analysis were ready after the patient had passed away.

### Molecular genetics

**Fig. 2:**
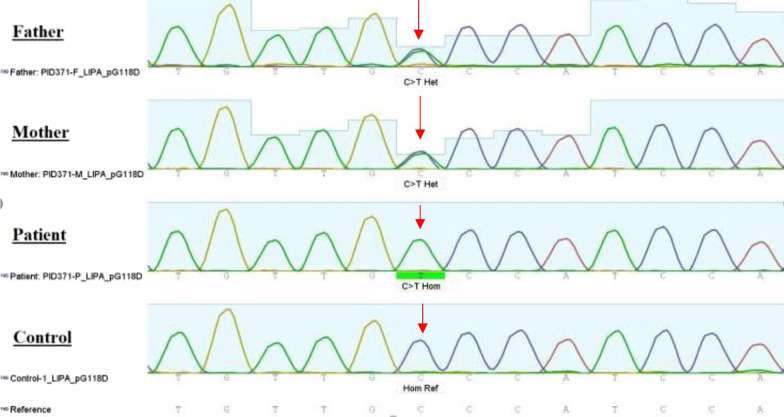
An electropherogram of father (top), mother (second), patient (third), and control (bottom), demonstrates a mutation (arrow) in exon 4 that results in conversion of amino acid glycine at position 118 to aspartic acid. On the top, the arrow indicates the paternally inherited c.482delA and the arrow in the second row approximates the location of the maternally inherited deletion

The whole exome sequencing (WES) process was performed with NovaSeq6000 Paired-end sequencing platform and read length of 150 bp paired-end (PE). The library type was SureSelect V6-Post and the coverage was 100×. The analysis of the WES data revealed a novel homozygous mutation in *LIPA* gene exon 4: NM_001127605:c. G353A (p.G118D), which converts the glycine amino acid to aspartic acid (Table 2). The parents’ heterozygous status for this variant was confirmed by Sanger sequencing method (Fig. 2).

**Table 2:** Features of the likely pathogenic variant found in our patient with Wolman disease presenting with HLH

	Gene	Zygosity	Variant	Inheritance	Outcome	MAF	OMIM	dbSNPrsID
Affected child	*LIPA*	Homozygous	G118D	AR	Likely pathogenic	< 0.01	613,497	–
Father	*LIPA*	Heterozygous	G118D					
Mother	*LIPA*	Heterozygous	G118D

The sequence analysis by in silico tool Polymorphism Phenotyping (PolyPhen) predicted that this missense mutation is probably damaging with a score of 1.000 (specificity of 100%). The same analysis with Protein Variation Effect Analyzer tools (PROVEAN) predicted a deleterious effect with PROVEAN score of −5.515 (cutoff: −2.5) [8–10].

### Search method

We searched PUBMED and SCOPUS for English language sources in April 2020, using the keywords HLH OR hemophagocytic lymphohistiocytosis and Wolman. All the found articles were read and the relevant ones were included. The search was repeated in July 2021 before final edition, and two articles was added to the main reviewed articles.

## Discussion and conclusions

In the light of our research in PubMed and SCOPUS for any case presentation of WD resembling manifestations of HLH, we found eight reports (see Tables 3 and 4). Perry *et al.* described three infants in 2001; a brother and sister presented at 49 and 26 days with hepatosplenomegaly, anemia, and elevated liver transaminases and triglycerides. With a diagnosis of familial erythrophagocytic lymphohistiocytosis, they were treated by chemotherapy and bone marrow transplant, respectively. Autopsy of the second infant revealed accumulation of cholesterol crystals and lipids in many organs, and a hypertrophied and calcified adrenal, leading to the diagnosis of WD following the finding of low acid lipase activity in liver biopsy. The third patient was a 25-day-old boy with hepatosplenomegaly, elevated liver transaminases and adrenal calcification. The patients died at a median age of 67 days [11].

**Table 3:** Comparisons of signs and symptoms in reviewed patients

Author	Sex	Age	Consanguinity	FTT	Fever	Diarrhea	Organomegaly	Death	Genetic variant
Perry *et al.* (2001) [11]	M (s)	49 days	Yes	–	–	–	Yes	67 days	–
	F (s)	26 days	Yes	–	–	–	Yes	67 days	–
	M	25 days	Yes	–	–	–	Yes	67 days	–
Santos *et al.* (2018) [4]	M	60 days	Yes	Yes	Yes	–	Yes	5 months	[*LIPA*] c.966 + 2T > G-intron 9 (in homozygosity)
	F	4 months	–	Yes	No	–	Yes	6 months	*LIPA* gene (c.509C > A (p.S103R)/c.796G > T(p.G266X),)
Taurisano *et al.* (2014) [3]	F	4 months	No	Yes	Yes	Yes	Yes	5 months	
Yavas *et al.* (2015) [13]	F	2 months	–	Yes	Yes	Yes	Yes	3 months	Exon 4 heterozygous location c:260G > T (GGC > GTC), p.Gly87Val
Elsayed *et al.* (2015) [14]	M	2.5 months	Yes	Yes	Yes	–	Yes	Not known	homozygous G969A (W130X) mutation
	F	3 months	Yes	Yes	Yes	–	Yes	Not known	homozygous c.438delC (p.S112X)
	M	3 months	Yes	Yes	Yes	–	Yes	Not known	c.G969A (p.W289X)
Tinsa *et al.* [15] (2018)	F	3 months	Yes	Yes	Yes	No	Yes	4 months	exon 3: NM_000235.3: c.153 C > A (p.Tyr51*)
Rabah *et al.* [16] (2014)	M	2 months	Yes		Yes	–	Yes	3 months	Not found
Alabbas *et al.* [17] (2021)	M	4 months	Yes	Yes	Yes	–	Yes	4 months	(428 + 1_967-1)_(*1_?)del in the *LIPA* gene (NM_000235.3; chr.10):
Present case (2021)	M	4.5 months	Yes	No	Yes	Yes	Yes	5 months	exon4:c.G353A:p.G118D, CADD: 32

**Table 4:** Comparisons of laboratory findings in reviewed patients

Author	Ferritin ng/ml	Coagulopathy	Hb (g/l)	platelet (× 10^9^/l)	WBC (× 10^6^/l)	Neutrophil (× 10^6^/l)	Bili T (mg/dl)	Bili D (mg/dl)	AST (U/l)	ALT (U/l)	LDH (U/l)	Albumin (g/dl)	Fibrinogen (mg/dl)	TG (mg/dl)	BM Hemophagocytosis
Perry *et al.* [11]	–	–	Low	–	–	–		–	Elevated	Elevated	–	–	–	Elevated	Yes
	–	–	Low	–	–	–		–	Elevated	Elevated	–	–	–	Elevated	Yes
	–	–	–	–	–	–		–	Elevated	Elevated	–	–	–	–	–
Santos *et al.* [4]	19,867		6.3	318,000	–	6120	Elevated	Elevated	299	68	3140	2.9	2.5	785	Yes
	21,577	Yes	8.4	117,000	–	8500	Elevated	Elevated	540	130	3300	1.9	3.18	539	Yes
Taurisano *et al.* [3]	8900	Yes	7.1	53,000	–	–	19.58	17.32	258	114	2302	2.4	1.24	450	Yes
Yavas *et al.* [13]	> 1650	–	7.6	92,000	12,000	–	–	–	–	–	–	–	–	361	Yes
Elsayed *et al.* [14]	2363	–	5	98,000	3800	–	–	–	–	–	1612	–	–	528	Yes
	1664	–	6.9	95,000	5600	–	–	–	78	17	1612	2.2	1.5	–	No
	2543	–	7.8	80,000	10,500	–	–	–			–	–	2	947	not done
Tinsa *et al.* [15]	4876	Yes	7.7	51,000	10,200	–	17	_	120	190	1107	–	1.8	308	Yes
Rabah *et al.* [16]	5600	Yes	6.9	73,000	11,900	–	19	13		38	3290	1.8	1.24	1594	Yes
Alabbas *et al.* [17]	22,683	Yes	6.6	39,000	4600	3200	19	9	670	229	–	3	1.6	–	Yes
Present case	5370	Yes	7.6	656,000	820	10	7.2	5.8	2930	603	2690	2.9	–	1110	–

*Hb* hemoglobin concentration, *WBC* white blood cell count, *Bili T* bilirubin total, *Bili D* bilirubin direct, *AST* aspartate transaminase level, *ALT* alanine transaminase level, *TG* triglyceride level, *BM* bone marrow

In 2018, Santos *et al.* presented two cases of Wolman disease. The first patient was a 2-month-old boy admitted with hepatosplenomegaly, fever, anemia, elevated liver transaminases, hypertriglyceridemia, and elevated ferritin level. Based on the criteria, the patient was treated according to HLH protocol [12]. Signs and symptoms subsided but recurred after a month. Peripheral blood smear showed lymphocyte cytoplasmic vacuolation and adrenal appeared calcified on X-ray. LAL activity in blood and fibroblasts was low. Later on, molecular PCR analysis revealed [*LIPA*] c.966 + 2T > G-intron 9 (in homozygosity), compatible with lysosomal acid deficiency. Sebelipase alfa was administered, which caused slight improvement, yet the patient died eventually with multiorgan failure. The second patient was a 4-month-old girl with hepatosplenomegaly, icterus, elevated liver transaminases, severe coagulopathy, hyperferritinemia, hypertriglyceridemia, and hypercholesterolemia. Hemophagocytosis in bone marrow and elevated CD25 were found. HLH therapy made no improvement. Further investigations showed a calcified adrenal and low LAL activity. Although sebelipase alfa was administered as a bridge therapy for HSCT, the patient died of cardiopulmonary compromise due to hepatic insufficiency. Molecular analysis of the *LIPA* gene identified two pathogenic mutations in compound heterozygous state: c.509C > A (p. S103R)/c.796G > T (p. G266X) [4].

Taurisano *et al.* in 2014 discussed a 4-month-old female case with hepatosplenomegaly, icterus, anemia, thrombocytopenia, elevated liver transaminases, and hypertriglyceridemia. Liver biopsy portrayed Kupffer cells with lysosomes containing crystals of cholesteryl esters. Bone marrow aspiration revealed giant histiocytic cells and signs of hemophagocytosis. Reduced LAL confirmed the diagnosis of WD. The patient went through supportive therapies, and died due to respiratory failure when she was 5 months old [3].

Yavas *et al.* reported a 2-month-old girl in 2015 with fever, diarrhea, failure to thrive (FTT), and hepatosplenomegaly. Anemia, thrombocytopenia, elevated liver transaminases and hypertriglyceridemia were also found. Hemophagocytic lymphohistiocytosis was observed in bone marrow aspiration. She was primarily diagnosed with HLH, then a diagnosis of WD was made because of low LAL activity. The patient died 1 month later, and molecular analysis revealed exon 4 heterozygous variation at the *LIPA* gene, location c:260G > T (GGC > GTC), p. Gly87Val [13].

Elsayed *et al.* in 2015 reported three patients with diagnosis of WD. The first patient was a 2.5-month-old boy with fever, hepatomegaly, anemia, and erythropoiesis in bone marrow aspiration. Hyperferritinemia, hypertriglyceridemia, and high LDH level were also observed. Sequencing of the *LIPA* gene demonstrated homozygous G969A (W130X) mutation which made the diagnosis of WD. The second patient was a 3-month-old girl presented with the same presentations as the first one. Vacuolated macrophages were found in bone marrow aspiration as well as histiocytosis. Sequencing of all coding sequences of the *LIPA* gene revealed homozygous mutation c.438delC (p.S112X) and led to the diagnosis of WD. The third patient, a 3-month-old boy, also with the same presentation and laboratory findings, went through sequencing of *LIPA* gene, which revealed homozygous mutation c. G969A (p. W289X), leading to the diagnosis of WD [14].

Tinsa *et al.* reported a 3-month-old girl in 2018 with fever and hepatosplenomegaly. Laboratory values were consistent with HLH disease. Bone marrow biopsy illustrated abnormal macrophages, but no evidence of hemophagocytosis. Three weeks later, adrenal calcification was found and bone marrow aspiration was repeated, revealing bubble-like cytoplasm in macrophages, compatible with Wolman disease. There was a novel homozygous mutation in *LIPA* gene exon 3: NM_000235.3: c.153 C > A (p. Tyr51*), which interrupted the reading frame by a premature STOP codon and confirmed the diagnosis of Wolman disease. The parents were heterozygous for this mutation [15].

Rabah *et al.* reported a 2-month-old boy in 2014, primarily diagnosed with HLH due to fever, hepatosplenomegaly, icterus, anemia, thrombocytopenia, elevated liver enzymes, hyperferritinemia and hypofibrinogenemia. Soap bubble-like macrophage cytoplasm in addition to hemophagocytosis were observed in bone marrow biopsy. Diagnosis of WD was made by leukocytic cholesteryl esterase assay, and molecular analysis showed no mutation [16].

Alabbas *et al.* reported a male infant in 2021, previously diagnosed with WD, but not treated due to unavailability of medication. He presented with secondary HLH at 4 months. They reported a novel mutation in *LIPA* gene; a deletion/duplication genetic analysis by real-time quantitative PCR (qPCR) confirmed the presence of homozygous deletion c. (428 + 1_967-1) _ (*1_?) del in the *LIPA* gene (NM_000235.3; chr.10): (OMIM 613,497) [17].

The discussed patients, including ours, consist of 14 cases. They aged from 25 to 135 days (median 90 days). Consanguinity was reported in nearly all of the cases. Hepatosplenomegaly was observed in all cases and fever was seen in most of them. Laboratory findings included anemia in all patients and thrombocytopenia, elevated liver enzymes, hypoalbuminemia, hypofibrinogenemia, and elevated LDH and triglycerides in most cases. Bone marrow hemophagocytosis was observed in 9 patients and adrenal calcification was reported in 8. Except for one report, which did not point to the patient’s survival, all the other patients were dead in less than 2 months from admission. A molecular analysis concordant with Wolman disease was found in 9 patients.

Witeck *et al.* conducted a systematic review in June 2020 in which they included all published articles on WD, finally incorporating 108 articles [18]. According to Witeck’s review, the median reported ages of the patients at onset and at diagnosis were 1.5 and 3 years, respectively. The most common clinical presentations were reported as hepatomegaly (93%), splenomegaly (77%), abdominal distension (52%), failure to thrive (66%), diarrhea (51%), vomiting (36%), and jaundice (8.2%). Laboratory values revealed anemia (55%), elevated transaminases (33%), low high-density cholesterol (37%), hypertriglyceridemia (22%), and hypercholesterolemia (8%). By meticulously studying features of patients presenting with HLH and keeping the important differential diagnosis of WD in mind, we can reach the correct diagnosis, which, by leading to initiation of the proper treatment, can save the patient’s life.

WD and CESD are two phenotypes of the same disorder in which there is low or absent LAL activity. CESD is presented by hyperlipidemia, atherosclerosis, and hepatic fibrosis, and has lower mortality than WD. CESD patients typically present missense mutations, correlating with some residual enzyme activity (5–10%). The mutation of c.894G.A in exon 8 is the most common mutation found in CESD patients, which comprises more than 50% of all reported variants [19]. Very low or absent LAL activity can be found in WD due to several dozens of mutations in the *LIPA* gene; that is deletions, insertions, and nonsense mutations [20].

**Fig. 3:**
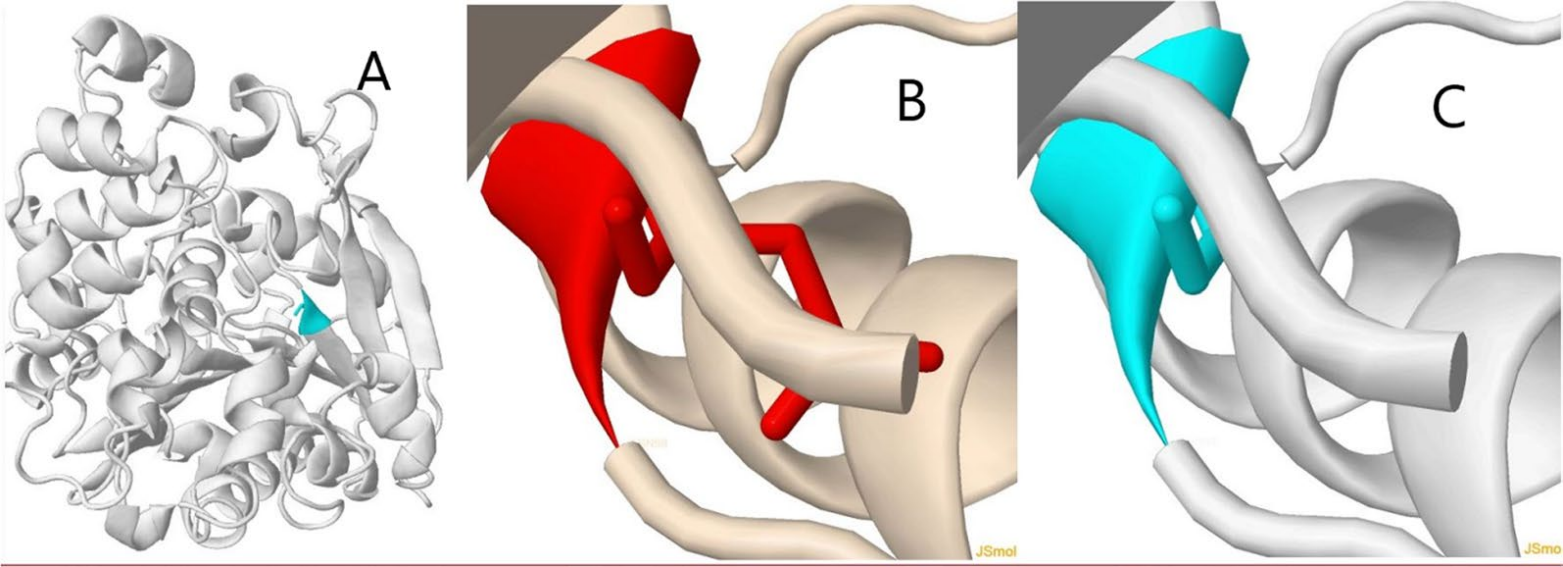
Three-dimensional illustration of the original *LIPA1* molecule with wild-type glycine residue (**A**) and a more detailed look inside the *LIPA1* molecule with wild-type (**B**) and the mutated residue (**C**). Considering the similarities in presentations of WD and HLH, the patient’s life can be saved if special attention is paid to presenting features of a patient with suspected HLH, that is symptoms, findings on physical exams, laboratory values, and radiologic findings, and if the proper treatment is urgently initiated. Reporting the novel mutations of WD can help geneticists interpret the results of their patients’ genetic studies appropriately, leading to correct diagnosis and treatment

The family that we are reporting presents a novel variant, caused by a glycine to aspartic acid substitution within exon 4 of the *LIPA* gene, which is predicted as likely pathogenic. This prediction was made using variant disease and population databases in silico tools (Fig. 3) and segregation analysis (Fig. 2) in the nuclear family and was thus presumed to be the cause of LAL-deficiency in this infant [21]. The literature was sought in regular time intervals and still, no description of this mutation was found.

The original wild-type residue (glycine) and newly introduced mutant residue (aspartic acid) each have their own specific size, charge, and hydrophobicity value. The wild-type residue charge is neutral compared with the negative charge of the mutant residue and is more hydrophobic than the mutant. The mutation imposes a charge which can repulse ligands or other residues with the same charge. The mutant residue is bigger than the wild-type and might lead to the formation of bumps. The torsion angles for this residue are unusual. Only glycine is flexible enough to make these torsion angles and mutation into another residue will force the local backbone to change into an incorrect conformation, which will disturb the local structure and impair the protein’s function. The original residue is conserved to a large extent, but a few other residue types have also been found at this position. Considering the conservation scores, this mutation can disrupt the protein’s function. The mutated residue is placed in a domain that plays a crucial role in the main activity of the protein and mutation of the residue can disrupt this function [21, 22].

In this case report, the causative point mutations result in the substitution of a glycine (the smallest amino acid) residue by a bulkier amino acid (aspartic acid) (Fig. 3). However, a loose correlation between the location of the point mutation along the molecule and disease severity exists for *LIPA1*. Previous research has revealed severely pathogenic and even lethal outcomes for glycine to aspartic acid substitutions within fibrillar collagen genes [23]. In our case, however, we are dealing with an enzyme molecule and not a structural protein; thus, these findings cannot be extrapolated to our case. Finally, we have to bear in mind that the context of the sequence surrounding the substitution, or mutations within special helical domains, significantly influence the effect of particular mutations.

## Data Availability

The datasets generated and/or analyzed during the current study include patient’s data; that is why they are not published online, but they are available from the corresponding author upon reasonable request.

## Abbreviations

WD: Wolman disease
HLH: Hemophagocytic lymphohistiocytosis
LAL: Lysosomal acid lipase
HSCT: Hematopoietic stem cell transplantation
NK: Natural killer
CESD: Cholesteryl ester storage disease
PT: Prothrombin time
PTT: Partial thromboplastin time
AST: Aspartate transaminase
ALT: Alanine transaminase
ALP: Alkaline phosphatase
WBC: White blood cells
FTT: Failure to thrive
Hb: Hemoglobin
TG: Triglyceride
BM: Bone marrow
PE: Paired-end

## References

[1] Madkaikar M, Shabrish S, Desai M (2016). Current updates on classification, diagnosis and treatment of hemophagocytic lymphohistiocytosis (HLH). Indian J Pediatr.

[2] Zhang JR, Liang XL, Jin R, Lu G (2013). HLH-2004 protocol: diagnostic and therapeutic guidelines for childhood hemophagocytic lymphohistiocytosis. Zhongguo Dang Dai Er Ke Za Zhi.

[3] Taurisano R, Maiorana A, De Benedetti F, Dionisi-Vici C, Boldrini R, Deodato F (2014). Wolman disease associated with hemophagocytic lymphohistiocytosis: attempts for an explanation. Eur J Pediatr.

[4] Santos Silva E, Klaudel-Dreszler M, Bakula A, Oliva T, Sousa T, Fernandes PC, Tylki-Szymanska A, Kamenets E, Martins E, Socha P (2018). Early onset lysosomal acid lipase deficiency presenting as secondary hemophagocytic lymphohistiocytosis: two infants treated with sebelipase alfa. Clin Res Hepatol Gastroenterol.

[5] Trottestam H, Horne A, Aricò M, Egeler RM, Filipovich AH, Gadner H, Imashuku S, Ladisch S, Webb D, Janka G (2011). Chemoimmunotherapy for hemophagocytic lymphohistiocytosis: long-term results of the HLH-94 treatment protocol. Blood.

[6] Aguisanda F, Thorne N, Zheng W (2017). Targeting Wolman disease and cholesteryl ester storage disease: disease pathogenesis and therapeutic development. Curr Chem Genom Transl Med.

[7] Pastores GM, Hughes DA (2020). Lysosomal acid lipase deficiency: therapeutic options. Drug Des Devel Ther.

[8] Adzhubei IA, Schmidt S, Peshkin L, Ramensky VE, Gerasimova A, Bork P, Kondrashov AS, Sunyaev SR (2010). A method and server for predicting damaging missense mutations. Nat Methods.

[9] Choi Y. A fast computation of pairwise sequence alignment scores between a protein and a set of single-locus variants of another protein. In: Proceedings of the ACM Conference on Bioinformatics, Computational Biology and Biomedicine: 2012; 2012: 414–417.

[10] Choi Y, Sims GE, Murphy S, Miller JR, Chan AP (2012). Predicting the functional effect of amino acid substitutions and indels. PLoS ONE.

[11] Perry R, Kecha O, Paquette J, Huot C, Van Vliet G, Deal C (2005). Primary adrenal insufficiency in children: twenty years experience at the Sainte-Justine Hospital, Montreal. J Clin Endocrinol Metab.

[12] Henter JI, Horne A, Aricó M, Egeler RM, Filipovich AH, Imashuku S, Ladisch S, McClain K, Webb D, Winiarski J (2007). HLH-2004: diagnostic and therapeutic guidelines for hemophagocytic lymphohistiocytosis. Pediatr Blood Cancer.

[13] Yavaş AK, Orhaner B, Genç P, Kılıç N, Erdoğan H, Özdemir Ö, Ekici A (2017). Secondary hemophagocytic lymphohistiocytosis in an infant with Wolman disease. Turk J Hematol.

[14] Elsayed S, Elsobky E, Tantawy A, Ragab E, Gil M, Lambert N, de Saint BG (2016). Wolman disease in patients with familial hemophagocytic lymphohistiocytosis (FHL) negative mutations. Egypt J Med Human Genet.

[15] Tinsa F, Ben Romdhane M, Boudabous H, Bel Hadj I, Brini I, Tebib N, Louati H, Bekri S, Boussetta K (2019). A novel mutation c. 153 C> A in a Tunisian girl with Wolman disease and unusual presentation: hemophagocytic lymphohistiocytosis. J Pediatric Hematol/Oncol.

[16] Rabah F, Al-Hashmi N, Beshlawi I (2014). Wolman’s disease with secondary hemophagocytic lymphohistiocytosis. Pediatr Hematol Oncol.

[17] Alabbas F, Elyamany G, Alanzi T, Ali TB, Albatniji F, Alfaraidi H (2021). Wolman’s disease presenting with secondary hemophagocytic lymphohistiocytosis: a case report from Saudi Arabia and literature review. BMC Pediatr.

[18] Witeck CR, Schmitz AC, de Oliveira JMD, Porporatti AL, De Luca Canto G, Pires MMS (2021). Lysosomal acid lipase deficiency in pediatric patients: a scoping review. Jornal de Pediatria.

[19] Fasano T, Pisciotta L, Bocchi L, Guardamagna O, Assandro P, Rabacchi C, Zanoni P, Filocamo M, Bertolini S, Calandra S (2012). Lysosomal lipase deficiency: molecular characterization of eleven patients with Wolman or cholesteryl ester storage disease. Mol Genet Metab.

[20] Reynolds T (2013). Cholesteryl ester storage disease: a rare and possibly treatable cause of premature vascular disease and cirrhosis. J Clin Pathol.

[21] Venselaar H, Te Beek TA, Kuipers RK, Hekkelman ML, Vriend G (2010). Protein structure analysis of mutations causing inheritable diseases. An e-Science approach with life scientist friendly interfaces. BMC Bioinf.

[22] Mustafa MI, Osman EA, Abdelmoneiom AH, Hassn DM, Yousif HM, Mahgoub IK, Badawi RM, Albushra KA, Abdelhameed TA, Hassan MA. Comprehensive in silico Analysis of IKBKAP gene that could potentially cause Familial dysautonomia. bioRxiv 2018:436071.

[23] Kuivaniemi H, Tromp G, Prockop DJ (1991). Mutations in collagen genes: causes of rare and some common diseases in humans. FASEB J.

